# Nanoscale 3D quantitative imaging of 1.88 Ga Gunflint microfossils reveals novel insights into taphonomic and biogenic characters

**DOI:** 10.1038/s41598-020-65176-w

**Published:** 2020-05-18

**Authors:** L. Maldanis, K. Hickman-Lewis, M. Verezhak, P. Gueriau, M. Guizar-Sicairos, P. Jaqueto, R. I. F. Trindade, A. L. Rossi, F. Berenguer, F. Westall, L. Bertrand, D. Galante

**Affiliations:** 1 Brazilian Synchrotron Light Laboratory (LNLS), Brazilian Center for Research in Energy and Materials (CNPEM), Av. Giuseppe Maximo Scolfaro, 10000, 13083-100, Campinas, Brazil; 2 Institute of Physics of São Carlos, University of São Paulo, Av. Trabalhador são-carlense, 400, 13566-590, São Carlos, Brazil; 3 Centre de Biophysique Moléculaire, CNRS, Rue Charles Sadron, 45071, Orléans, France; 4 Dipartimento di Scienze Biologiche, Geologiche e Ambientali (BiGeA), Università di Bologna, via Zamboni 67, I-40126, Bologna, Italy; 5 Paul Scherrer Institut, Forschungsstrasse 111, 5232, Villigen, Switzerland; 6 Université Paris-Saclay, CNRS, ministère de la culture, UVSQ, IPANEMA, 91192, Saint-Aubin, France; 7 Department of Geophysics, Institute of Astronomy, Geophysics and Atmospheric Sciences, University of São Paulo, Rua do Matão, 1226, 05508-090, São Paulo, Brazil; 8 Brazilian Center for Research in Physics (CBPF), R. Dr. Xavier Sigaud, 150, 22290-180, Rio de Janeiro, Brazil; 9 Synchrotron Soleil, Saint-Aubin, L’Orme des Merisiers, BP 48 Saint-Aubin, 91192, Gif-sur-Yvette, France; 10 Université Paris-Saclay, 91190, Saint-Aubin, France; 11 Present Address: ISterre, UGA, CNRS, Observatoire des Sciences de l’Univers, CS 40700, 38058, Grenoble, France; 12 Present Address: Institute of Earth Sciences (ISTE), University of Lausanne, Lausanne, Switzerland

**Keywords:** Biogeochemistry, Physics

## Abstract

Precambrian cellular remains frequently have simple morphologies, micrometric dimensions and are poorly preserved, imposing severe analytical and interpretational challenges, especially for irrefutable attestations of biogenicity. The 1.88 Ga Gunflint biota is a Precambrian microfossil assemblage with different types and qualities of preservation across its numerous geological localities and provides important insights into the Proterozoic biosphere and taphonomic processes. Here we use synchrotron-based ptychographic X-ray computed tomography to investigate well-preserved carbonaceous microfossils from the Schreiber Beach locality as well as poorly-preserved, iron-replaced fossil filaments from the Mink Mountain locality, Gunflint Formation. 3D nanoscale imaging with contrast based on electron density allowed us to assess the morphology and carbonaceous composition of different specimens and identify the minerals associated with their preservation based on retrieved mass densities. In the Mink Mountain filaments, the identification of mature kerogen and maghemite rather than the ubiquitously described hematite indicates an influence from biogenic organics on the local maturation of iron oxides through diagenesis. This non-destructive 3D approach to microfossil composition at the nanoscale within their geological context represents a powerful approach to assess the taphonomy and biogenicity of challenging or poorly preserved traces of early microbial life, and may be applied effectively to extraterrestrial samples returned from upcoming space missions.

## Introduction

Understanding Precambrian fossilized microorganisms, where preserved, can provide critical insights into the earliest records of life on Earth and its paleoenvironment^1–6^, especially in light of controversies surrounding the origin of chemical biosignatures such as isotopic fractionation^7,8^ and biomolecules^9^. Nonetheless, imaging the morphologies of these micrometric structures demands high spatial resolution, while the composition of fossilized cellular remains imposes stringent limitations in terms of contrast and penetration depth of the probing radiation, thereby introducing technical constraints for the evaluation of the earliest records of life at cellular to sub-cellular levels. The importance and relative rarity of most Precambrian fossils entails using non-destructive methods, a significant constraint that is shared by efforts to develop approaches for the search for potential fossil biosignatures in samples returned from Mars in the near future^10,11^.

The combination of distinct high-resolution analytical methods such as transmission and scanning electron microscopy (TEM and SEM), synchrotron X-ray spectroscopic techniques such as scanning transmission X-ray microscopy (STXM) and other spectroscopic analyses complemented by confocal microscopy (e.g. micro-Raman and confocal laser scanning microscopy) have significantly advanced our comprehension of Precambrian microfossils in the last decades^12–17^. Retrieving 3D morphological and geochemical information is a crucial addition to unequivocally resolve the nature of controversial structures which could be misinterpreted when analyzed using only two-dimensional sections. Nano-tomography using a focused ion beam coupled to SEM (3D-FIB-SEM) is currently the main technique used for the nanoscale assessment of microfossils in three-dimensions^18,19^. Its limitations for the study of early life are primarily due to its destructive nature and high cost, the latter shortcoming adding to restrictions on the sampling volume and resolution, which are also practically limited by the thickness of the slices (usually around 100–200 nm^17^).

Herein, we explore the potential of ptychographic X-ray computed tomography (PXCT)^20,21^ as a step towards solving such limitations in the study of Precambrian microfossils by providing high penetrability and non-destructive 3D assessments that are impossible using electron microscopy, coupled with quantitative contrast analysis of phases at the nanoscale. Ptychography is an X-ray coherent diffraction imaging technique based on measuring a series of far-field diffraction patterns at a set of overlapping sample areas and application of computational phase retrieval algorithm to retrieve the reconstructions of the sample phase and absorption simultaneously^20,21^. In PXCT, the individual projections are acquired and reconstructed at each tomographic angle to obtain 3D volume. PXCT results in the 3D electron density maps of structures over a field of view of tens of micrometers with nanometric resolution. PXCT thus fills a crucial gap in resolution and field-of-view between TEM and microscale imaging methods, allowing for example the imaging of whole extant cells in 3D with resolution and contrast for identifying organelles without staining^22^. For geological materials, the quantitative determination of electron density can be used to characterize minerals and organic compounds *in situ* with high precision^23,24^, thus presenting novel insights on the mechanism of preservation and taphonomic history of the specimen.

**Figure 1:**
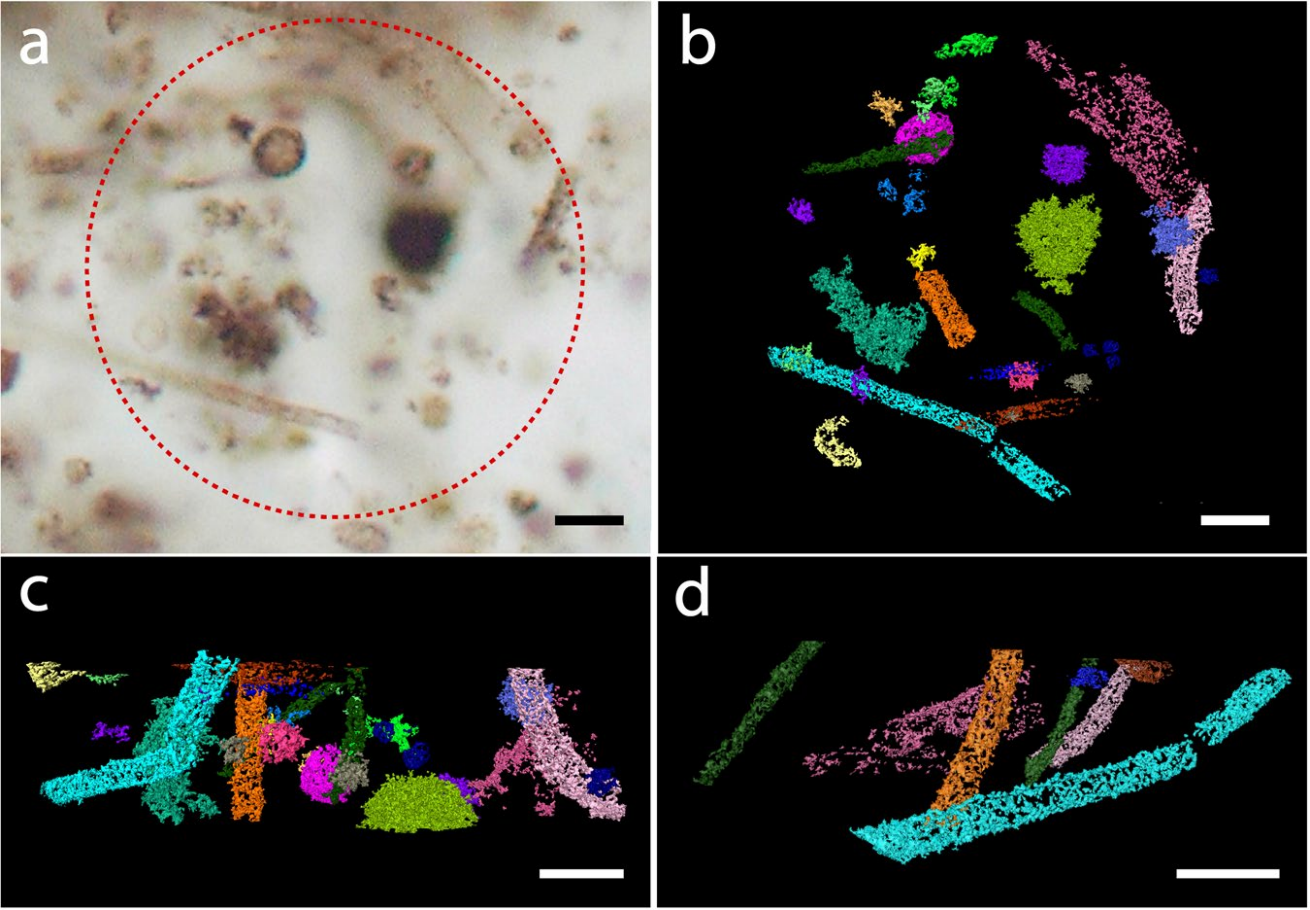
Fossilized microorganisms from the Schreiber Beach locality. (**a**) Optical photomicrograph of kerogenous microfossils. The circle indicates the region selected for PXCT analysis. (**b**) 3D rendering of the fossils distinguished in the PXCT data. (**b)** Is at the same magnification and approximately in the same orientation as **a**. (**c**) Lateral view of the sample showing microfossils within the 10 µm high sample analyzed using PXCT. (**d**) 3D rendering of *Gunflintia* filaments in the sample. Scalebars: 5 μm.

We have imaged microfossils from the Schreiber Beach and Mink Mountain localities of the Gunflint Formation (1.88 Ga, Ontario, Canada), which present remarkably different assemblages and states of preservation of fossilized biota. Schreiber Beach material contains exceptionally well-preserved carbonaceous specimens with conserved cellular outlines composed of kerogenous material that have been extensively studied by several imaging methods^15,16,18,25^. In contrast, specimens from Mink Mountain, which suffered a higher degree of subsequent alteration and metamorphism are poorly-preserved and have thus received relatively limited attention^15,26,27^. The Mink Mountain specimens are described as carbon-poor microfossils, where cellular remains have been substituted by the iron-oxide hematite^26,27^. In this work, we focused on the comparative evaluation of filamentous microstructures (Fig. 1A) interpreted as *Gunflintia*^28^ and present in samples from both localities.

## Results

We obtained a pixel size of 28.53 nm and spatial resolutions of 59 nm and 52 nm for the Schreiber Beach and Mink Mountain samples, respectively (see Supplementary Fig. S1 and Material and Methods for explanations about resolution estimation). These resolutions and the high contrast allowed unprecedented investigation of the three-dimensional ultrastructure of these specimens (Supplementary Videos S1 and S2) and revealed remarkably different taphonomic histories for the fossils from each locality. The quantitative electron density contrast implies that, in the 2D tomographic virtual slices, the pixel intensity is related to the electron density, *i.e*. features that appear darker in the virtual slices present lower electron densities than those appearing brighter.

### Schreiber beach locality samples

**Figure 2:**
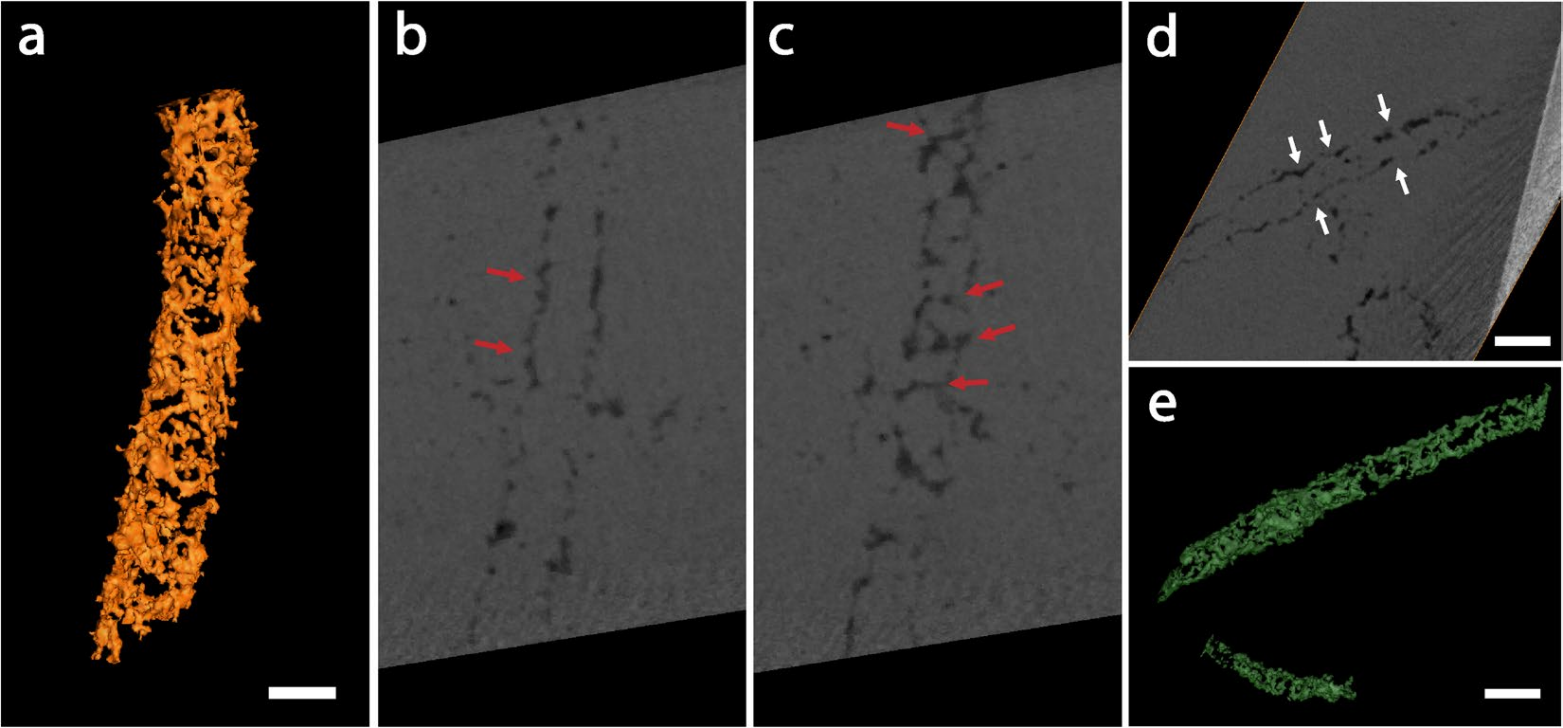
Selected filaments from the Schreiber Beach sample. (**a**) 3D rendering showing a discontinuous, irregular cell wall. (**b**) Detail of a longitudinal section from the center of the specimen showing an irregular cell wall morphology with saw-tooth-like morphology (red arrows), but no inner traces of septation. (**c**) Off-centered longitudinal section close to the border of the filament showing an inner “septa-like” pattern (red arrows) that in fact represents a taphonomic remobilization of sheath material. (**d**) Transversal 2D view of another filament showing periodic constrictions indicative of septation (white arrows) and (**e**) 3D rendering of this specimen. Note adjacent spheroidal microfossil in (**d)** also showing the 2D saw-tooth pattern of carbonaceous material. Scale bars: 1 μm.

Schreiber Beach microfossils comprise diverse morphotypes (Fig. 1), including filaments (*Gunflintia* species), spheroids (e.g., *Huroniospora* species) (Supplementary Fig. S2), and other complex morphologies that are similar to *Eosphaera* (Supplementary Fig. S3), even though with smaller dimensions than most examples of this morphotype (~5 μm). All fossil morphotypes present thin, irregular and discontinuous kerogenous cell walls, with 2D “saw-tooth” patterns indicative of the rearrangement of kerogenous material during the recrystallization of microcrystalline silica into quartz crystals (Fig. 2A–C, Supplementary video S1). These results are consistent with, and similar to previous observations using 3D-FIB-SEM and TEM^17–19,25^. Although septation can be preserved in some *Gunflintia minuta* specimens^16^, no inner traces of septa were observed in any of the tubular specimens. The discontinuous texture caused by remobilization of the tubular sheath material^16^, when observed in off-centered 2D cross-sections, *i.e*., close to the surface of the specimen, can display morphologies that could, in 2D, be misinterpreted as septation (Fig. 2C), a classical biogenicity character^4,6^. This illustrates one important aspect of 3D imaging for the unambiguous evaluation of morphological and taphonomic characters in fossils that can be important for biogenicity attestations. The fact that no septation was observed could either (i) reflect the absence of septation in all studied filaments, (ii) result from the degradation of septa during taphonomic process, or (iii) result from resolution limitations if their dimensions are significatively smaller than the spatial resolution. One of the studied filaments, rendered in green (Fig. 2D,E), presents periodic transversal constrictions similar to the morphology of previously described septate specimens^16,17^, where the septa insertion is associated with a periodic constriction of the cell envelope. This filament is only approximately 0.90 μm in width, thus inner traces of septae, if preserved, would likely be too thin to be resolved with our spatial resolution. Periodic constrictions observed in 3D could nevertheless be useful indicators of septation when such structures are not visible.

No other minerals or materials denser than the matrix were observed to be associated with filaments in the Schreiber Beach sample. The mass density of the matrix was estimated to be 2.68 ± 0.08 g/cm^3^, consistent with quartz (2.66 g/cm^3^). The density of the kerogenous material, measured in the inner regions of larger structures to avoid contributions to the signal from the surrounding material (see Discussion, Methods and Fig. S4 for details) is estimated as 1.39 ± 0.18 g/cm^3^. This density is consistent with thermally mature kerogen (1.19 g/cm^3^ to 1.77 g/cm^3^)^29–32^ as expected from the dark brown color of these structures when observed using optical microscopy^5^.

### Mink mountain locality samples

**Figure 3:**
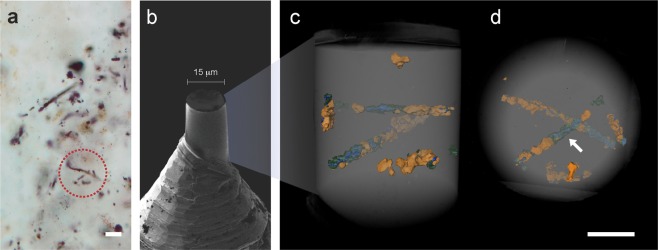
Fossilized filamentous microorganisms from the Mink Mountain locality viewed by different imaging methods. (**a**) Optical photomicrograph of filamentous microfossils apparently preserved as a red-brown iron oxide. The circle indicates the region selected for PXCT analysis. (**b**) SEM micrograph of pillar sample prepared by FIB-SEM attached atop a PXCT pin. (**c,d**) 3D renderings (side and top views, respectively) of PXCT data showing the distribution of fossil filaments in the interior of the pillar. The view in (**d)** corresponds to the same orientation as the outlined region shown in (**a**). The white arrow indicates one of the kerogenous regions (green/blue) that is invisible in the optical microscopy shown in (**a**). The structures in orange represent the only visible features resolved by optical microscopy and correspond to euhedral and anhedral maghemite crystals. Scale bars: 5 μm.

**Figure 4:**
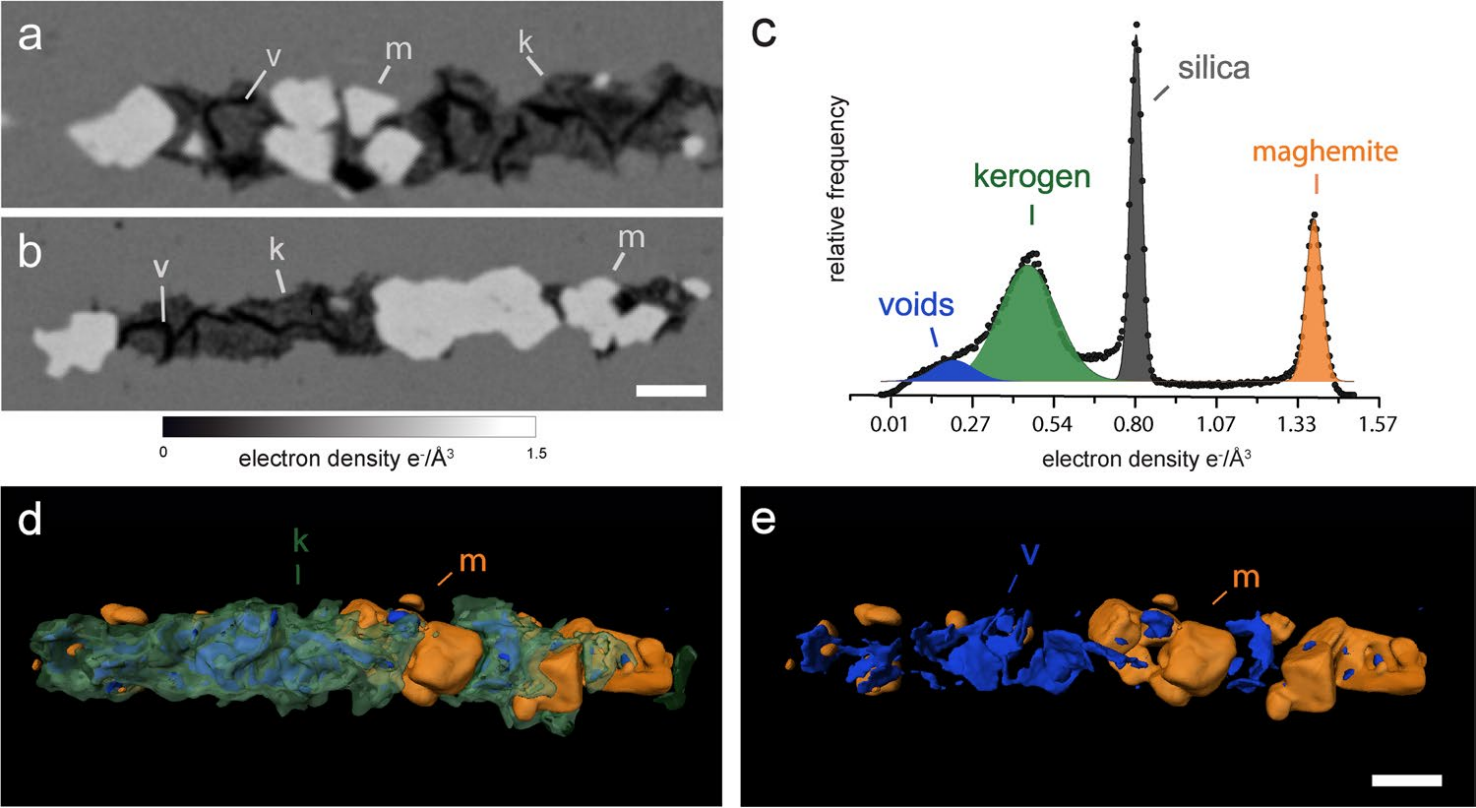
PXCT results for fossil filaments from the Mink Mountain locality. (**a,b**) Details of tomographic virtual slices showing two different fossil filaments within the silica matrix, mostly composed of an irregularly distributed kerogenous material (k). Voids (v) are visible within the kerogen, indicating a high maturity and brittle behavior characteristic of thermal cracking during the generation of fluids and gases. The irregular aspect of the kerogen probably results both from recrystallization of microquartz and brittle deformation with crack formation. The outline of quartz crystals that displaced the kerogen can be observed in the right side of the filament shown in (**a**). Higher density maghemite (m) is present as euhedral and anhedral crystals. (**c**) Histogram of electron density and Gaussian fits obtained from a representative volume of the sample containing the four different phases. The centers of the Gaussians were used for quantitative analysis and identification of the compounds (see Methods). (**d**) 3D detailed segmentation view of a filament showing maghemite crystals with cubic and octahedral morphologies, and kerogen (semi-transparent green) with interior cracks. (**e**) Internal view of the kerogenous region in (**d**) showing the distribution and shape of the cracks (blue). Scale bars: 1 μm.

Specimens from the Mink Mountain locality are less well preserved than those from Schreiber Beach and have more complex compositions, including features detectable using PXCT that could not be previously resolved by optical microscopy. Three distinct material phases were identified (Figs. 3 and 4, Supplementary video S2), for which mass densities were established from the PXCT and compared to the tabulated value (Table 1). The density of the matrix (2.69 ± 0.03 g/cm^3^) is also consistent with quartz (2.66 g/cm^3^), as expected from previous studies^26^. The filamentous morphology of the microfossils is consistent with *Gunflintia sp*, with an average diameter of 1 μm and variable length. Filaments are mainly composed of an irregularly shaped, discontinuous and non-hollow material of low density (green in Figs. 3C,D, and 4D, dark-grey in Fig. 4A,B). Despite being the major component of the specimens, this material is not resolved using optical microscopy (Fig. 3A,D). It presents a range of mass densities centered on 1.50 ± 0.18 g/cm^3^, consistent with thermally mature kerogen but inconsistent with both amorphous carbon and graphite (>2 g/cm^3^), compounds that can be misinterpreted as biogenic carbonaceous material by Raman analysis alone. Besides the difference in morphology compared to the filaments from Schreiber Beach, the density of this kerogenous material is also higher, *i.e*. suggesting higher diagenetic temperatures, consistently with estimations from previous studies^15^. The thermal maturation of kerogen can also lead to its cracking and the generation of fluids and gases when it develops a characteristic brittle behavior^32,33^. The low-density cracks observed within the kerogenous masses (blue in Figs. 3C,D, 4D,E) are consistent with the presence of fluids/gases released during the thermal cracking of mature organic material. The morphological irregularity of kerogen (Fig. 4A,B,D) observed at the micro- and nanoscale indicates the displacement of this material due to the recrystallization of microcrystalline silica during late diagenesis and low-grade (zeolite to sub-greenschist) metamorphism^25^.

**Table 1:** Phases of compounds identified in the Mink Mountain filaments on the basis of their calculated mass density from obtained refractive indices, and comparison with reference values^26,27,35,50^.

Phase	Mass density (g/cm^3^)	Material	Reference mass density (g/cm^3^)
*Silica matrix*	***2.68*** ± ***0.03***	*Quartz*	***2.66***
*Kerogenous material*	***1.50*** ± ***0.18***	*Mature kerogen*	***1.25–1.40***
*Maghemite*	***4.82*** ± ***0.08***	*Hematite*	***5.24***
		*Magnetite*	***5.18***
		*Maghemite*	***4.87***

In some regions of the filaments, higher density iron oxide crystals are observed (white in Fig. 4A,B, orange in Figs. 3C,D, 4D,E), distributed heterogeneously. This mineral is the only phase presenting significant contrast in optical microscopy (Fig. 3A), and this has likely sustained the current interpretation of the Mink Mountain fossilization process as iron-substitution^26,27^. No rims or variation patterns in density are observed within the crystals (Fig. 4A,B). Their calculated density of 4.82 ± 0.08 g/cm^3^, however, is considerably lower than the densities of both hematite (5.24 g/cm^3^), the mineral previously described in these structures^26,27^ and magnetite (5.18 g/cm^3^; Table 1). This density instead matches the less common iron oxide maghemite (γ-Fe_2_O_3_), a possible weathering product of magnetite or of heating of other iron oxides that almost always adopts the habit of its precursor^34^. Previous taphonomic models for the iron-replaced Gunflint biota^27^ proposed that magnetite could result from ferrihydrite transformation in the presence of organics and, in higher metamorphic grades (amphibolite), could be secondarily oxidized to hematite. Other studies of Mink Mountain fossils detected hematite using bulk X-ray diffraction and, based on the octahedral morphology of the crystals, interpreted it as octahedral pseudomorphs of hematite after magnetite^26^. Based both on the density and the 3D evaluation of crystals, the latter of which revealed octahedral and cubic morphologies (Fig. 4D,E, Supplementary Video S2), we interpret this iron oxide as maghemite, the product of the transformation of magnetite, which in turn was formed from ferrihydrites in contact with the organics of the fossils^27,34^. Since the Mink Mountain locality experienced only sub-greenschist metamorphic grade, magnetite could not have been converted to secondary hematite as in the highest-grade zones of the Gunflint Formation^27^. To confirm the presence of magnetic phases, rock magnetic analysis – anhysteretic remanent magnetism (ARM) and isothermal remanent magnetism (IRM) – were performed in sections of the sample from which our PXCT sample was extracted. These results indicated a secondary magnetic phase after hematite, consistent with the presence of maghemite or magnetite (Supplementary text and Fig. S5 and S6). Raman point spectroscopy in adjacent filaments of the thin-section from which the PXCT sample was extracted showed only hematite peaks (Fig. S7). We conclude that the lack of maghemite peaks can be explained by the photochemical conversion of this mineral to hematite even with low laser powers in pure standards^35,36^. The low concentration of this mineral, summed to the possible presence of widespread fine-grained hematite^27^ could have contributed for the non-detection of maghemite Raman peaks, a fact already reported in previous works^37^. The Raman spectra also shows no signal of epoxy, discarding the possible contamination by this material in our sample.

## Discussion

Schreiber Beach samples, which are composed of thin, discontinuous carbonaceous cell envelopes (sheaths or cell walls) immersed in a silica matrix are among the most challenging structures for imaging methods in terms of their minimal contrast against the matrix phase. When structures have dimensions below the pixel size of the dataset, they are not resolved since each 3D pixel (voxel) comprises a mixture of kerogenous material and the surrounding silica. This also occurs at the border or interface of different materials and generates an intermediate pixel intensity, depending on the proportion of each material. This phenomenon, called *partial-volume effect*, has implications for the quantification of electron density and spatial resolution (See Material and Methods and Fig. S4 for a detailed discussion). Nonetheless, resolving such structures with PXCT constitutes an important advancement for the 3D imaging of Precambrian microfossils and shows that this approach is applicable to the investigation of a range of other challenging carbonaceous structures. The spatial resolution achievable will depend on several factors related to the sample composition, such as the complexity of structures present, and the contrast of the different phases present in the sample.

Two key findings of this work are i) a new interpretation of the complex taphonomic processes involved in the preservation of microfossils from the Mink Mountain locality and ii) highlighting the unique taphonomy of microfossils from this outcrop compared with the typical preservation of carbonaceous microfossils from other Gunflint Chert localities, such as the Schreiber Beach material described herein, where carbonaceous material forms cell walls or sheaths (Fig. 2)^16,18,25^. The detection of maghemite indicates that the presence of the organic material – in this case of biogenic origin – and its maturation into kerogen after the percolation of the iron-rich fluids drove a localized taphonomic pathway for iron oxides at the micrometric scale. This pathway is markedly different from the taphonomical processes of the locality in general, where organic matter was present in lower concentrations, resulting in hematite as the dominant mineral and hindering the detection of maghemite by conventional techniques, such as bulk X-ray diffraction^26^. In addition, the *in situ* observation of this iron-oxide pathway emphasises that this original strategy in 3D quantitative imaging has the potential to unveil cryptic taphonomic processes at the micro- and nano-scales that are irresolvable using classic petrographic and petrological indicators.

The integrated characterization of the 3D morphology and mineralogical composition of these fossils at the nanoscale leads to complementary means of assessing the biogenicity of microfossils in general. For instance, the presence of kerogen following the morphology of a cell wall or a membrane-like structure that delimits a hollow lumen, such as observed in the Schreiber Beach specimens, is widely considered a key character attesting to the biogenicity of fossilized microorganisms^13,14,25,38^. In poorly-preserved specimens, such as those from Mink Mountain, however, these fine morphological features are frequently not retained. In this context, the discrimination of thermally mature kerogen from abiotic carbonaceous material – such as crystalline graphite or the condensed Fischer Tropsch-type carbonaceous material formed in hydrothermal settings – is a critical issue^7,14^. In our Mink Mountain species, the homogeneous diameter of these kerogenous features at the micron-scale both in individual fragments of the filaments and throughout their full extent in three dimensions is consistent with a biogenic origin in the cell envelope of the organism^38^. This type of morphometric consistency is one example of a biogenicity criterion that can be important for the evaluation of poorly preserved specimens of disputed biogenicity and can be unequivocally attested using 3D nanoscale imaging over a volume of several cubic micrometers with PXCT.

Evaluating the mass density and 3D distribution of carbonaceous material both at the nano- and micro-scales provides an essential clue in the search for consistency between an original biotic morphology and the local taphonomical history. The possibility to observe the influence of microfossil diagenesis on local mineral formation pathways also permits the probing of microbe-mineral interactions that could help supporting or overturning the biogenicity of poorly preserved, potentially biological, microstructures. Furthermore, this could potentially assist the search for mineralogical biosignatures and an improved understanding of their genetic processes, which are often poorly constrained^39^.

The fact that the kerogenous material of some Mink Mountain specimens could not be observed by optical microscopy – the fundamental method of survey in the study of microfossils – further implies that contrast limitations of visible light microscopy may have led to shortcomings in the evaluation of carbonaceous microfossils and consequently in the demonstration of their biogenicity in certain lithological or petrological contexts. The analysis of kerogenous microfossils in three dimensions is challenging, even using high-resolution lab-based imaging techniques, particularly in Precambrian cherts where density contrasts against the impregnating silica matrix are very low^40^. This work further raises the need for a re-evaluation of controversial structures and a refinement of classical, but potentially outdated, biogenicity criteria (e.g.^38^) using novel methodological approaches with robust capabilities to discriminate kerogen in 3D and attest that both morphology, mineralogy and local taphonomy at the ultrastructural scale correspond to expectations for biogenic structures. Moreover, the geochemical characterization of the surrounding minerals will enable an accurate interpretation of the palaeoenvironmental setting, petrological context, and taphonomic and diagenetic histories of putative ancient biosignatures that may add confirmation of their singenicity with the host rock.

## Conclusion

Ptychographic X-ray computed tomography appears to be a highly promising non-destructive approach for the *in situ* assessment of the organic and mineral components of microfossils embedded in rock matrices, circumventing some of the limitations of electron microscopy and preserving fossils intact for further complementary analysis. Although it is possible to achieve higher observation resolutions in TEM, PXCT fills some of the gaps inherent in the 2D character of TEM by allowing a larger sampling volume (limited to approximately 100 nm thickness for high-resolution TEM) and providing three-dimensional continuity with electron density measurements; moreover, TEM sample preparation is typically destructive. Possible alteration due to radiation damage can be tracked during the scan and was not observed in our study (Supplementary Fig. S8). At present, the main practical limitations of this imaging method are the invasive requirement of sampling the rock pillar for analysis and the time frame required to prepare and scan samples. This restricts PXCT to the characterization of specific specimens rather than as a generalized exploratory technique.

The quantitative 3D insights into the nanoscale ultrastructure of microfossils within their geochemical and taphonomical contexts made possible using PXCT demonstrate its potential for evaluating and revisiting the biogenicity of ancient microfossils, particularly poorly preserved specimens. The unique possibility of interrogating biogenic characters at the nanoscale can measurably contribute to the debate surrounding the biogenicity of the most challenging and controversial morphological biosignatures from Precambrian rocks, including crucial reassessments of the earliest putative traces of life on Earth. On the longer term, this approach shows particular promise for the analysis of microscopic features of interest in samples returned by Mars missions.

## Methods

### Sample preparation

The Schreiber Beach sample (GF55) was collected by Andrew H. Knoll and prepared for optical petrography at the CNRS Orléans. FIB-milled cylinder of 25 μm diameter was extracted using a TESCAN LYRA3 Gallium FIB/SEM, at the LABNano from the Centro Brasileiro de Pesquisas Físicas (CBPF).

Mink Mountain *Gunflintia sp*. filaments were identified by optical microscopy (Olympus BX-51, CNRS Orléans) in petrographic thin sections from microfossil-rich regions that were extracted from thin sections of a filament-rich zone on the limb of a pseudocolumnar stromatolitic macrostructure (Supplementary Fig. S9). This sample (07CA22) was collected from the Mink Mountain locality, Ontario, Canada, by F. Westall in 2007. The sample location was recorded using Global Positioning System (GPS) coordinates (N 48°14'54,0” W 90°09'32,1”). The sample for PXCT was prepared as FIB-milled cylinder of 15 µm diameter using a Zeiss NVision 40 Gallium FIB/SEM at ScopeM, Zürich (Fig. 3B).

Both samples were mounted on PXCT flOMNI tomographic pins^41^.

### Ptychographic X-ray computed tomography

PXCT was carried out at the cSAXS beamline of the Swiss Light Source (SLS) at the Paul Scherrer Institut, Villigen, Switzerland, using the flOMNI setup described in detail elsewhere^21,42^. We used a photon energy of 6.2 keV and the sample were placed downstream of the focal spot; the beam at the sample position had a diameter of approximately 4.0 μm. Ptychographic scans were performed with a 22 × 15 μm field of view for the Mink Mountain sample and 44 × 10 μm for the Schreiber Beach. The scan points were positioned following a Fermat spiral trajectory^43^ with a step size of 1.2 μm and an exposure time of 100 ms per point. Ptychographic projections were reconstructed in an area of 300 × 300 pixels of the Pilatus detector placed 7.362 m downstream of the sample, resulting in a pixel size of 28.53 nm using 600 iterations of the difference map (DM) algorithm^44^ followed by 400 iterations of a maximum likelihood (ML) refinement^45^. For tomography, 1100 projections were recorded for Mink Mountain and 653 for Schreiber Beach, both equally spaced over a 180-degree angular range The phase of the reconstructed projections was used after post-processing alignment and removal of constant and linear phase components^46,47^, and a modified filtered back projection algorithm was applied for the tomographic reconstruction. The 3D half-period resolution was estimated by Fourier shell correlation (FSC) with the ½-bit threshold criterion. For this we split the projections into two independent datasets, each with double angular spacing, by taking either the even or odd angular projections. With this we then compute two independent tomograms. The FSC between these two tomograms is calculated and the point where the FSC intersects the 1/2 bit threshold defines the resolution (Supplementary Fig. S1)^21,48^.

### Tomographic data treatment

Segmentation, 3D rendering and analysis were performed using Avizo 9.5 (Thermo Scientific). The intensity of the pixels was the major criterion for the segmentation and no filter was applied to the images to maintain their original electron density infomation. For electron density quantification, a different segmentation was performed in order to avoid pixels at the border of material phases, which may contain a mixture of contributions from the studied object and its surroundings. For this, after thresholding segmentation, segmented selections were shrunken by 3 pixels in every direction. This shrinking value was estimated by considering the minimum size of a spherical particle of radius R that present a number of voxels within the volume (*n*_*V*_) higher than within its surface area (*n*_*A*_), *i.e*. 4π/3(*R*^3^/*l*) > 4π (*R*^2^/l), therefore *R* > *3* *l*, where *l* is voxel width. The segmented materials were exported as TIFF masks which were used for the extraction of the intensity histogram using the Fiji software.

### Mass density quantification

The pixel values of the phase tomogram provide the real part δ**r** of the complex refractive index of the sample, *n(****r****)* = δ*(****r****)* + *iβ(****r****)*. The electron density n_e_(**r**) was retrieved from the δ**r** values using the relation *n*_*e*_*(****r****)* = 2πδ(***r***)*/λ*^2^*r*_0_, where r_0_ denotes the classical electron radius and λ the wavelength of the radiation, as described in detail in Diaz *et al*.^22^.

After the segmentation of the material phases and extraction of intensity histograms with Fiji software, Gaussian functions were made using the MagicPlot 2.8 software (Magic Plot Systems, LCC). The peak center of each Gaussian function was then used to estimate center distribution of the electron density of each material, including air for normalization. In all cases, the range of distribution was taken as the standard deviation of the fitted Gaussian functions.

Mass density was estimated based on the electron density using the formula *ρ* = *n*_*e*_*A/N*_*A*_*Z*, where *A* is the molar mass, *Z* is the number of electrons and *N*_*A*_ is Avogadro’s number. We used the *A* and *Z* values of the candidate minerals^26,27,34^. For quartz (SiO_2_) *A/Z* = 2, and for hematite/maghemite and magnetite (Fe_3_O_4_, Fe_2_O_3,_), *A/Z* = 2.1. For kerogen, we used a composition of C_175_H_102_O_9_N_4_S_2,_ estimated by Ungerer, Collell, and Yiannourakou 2015^49^, which results in a ratio of *A/Z* ratio of 1.92 and is in agreement with typical values for this ratio in other biological compounds^22^. The calculated mass densities were compared with mineral candidates using the density values found in the mineral database *Mindat.org*.

### Magnetic analysis

Experiments were performed in a 2 G Enterprises model 755 superconducting quantum interference device (SQUID) with in-line alternating field coils and direct field (DC) in z-axis. The SQUID magnetometer is housed in a magnetically shielded room (magnetic intensity lower than 200 nT) and nominal sensitivity of 10^−12^ Am². For anhysteretic remanent magnetization (ARM), we used an alternating field of 100 mT and a direct field of 0.1 mT, whereas a stepwise isothermal remanent magnetization (IRM) with a maximum field of 1.2 T was obtained in a Vibrating Sample Magnetometer (VSM). The magnetic domain states were obtained with the first-order reversal curve (FORC) diagrams in a VSM, with maximum field of 1.4 T and smoothing factor of 6, the data was processed using the software FORCinel^50^.

### Raman spectroscopy

Raman point spectra were collected using a Renishaw (Renishaw PLC, Wotton-under-Edge, UK) inVia micro-Raman Spectrometer with HeNe laser line (633 nm), a 50× objective and CCD detector. Spectra were acquired at 5% and 10% laser power (850 µW and 170 µW of theoretical power) and 2 s exposure time, 200 accumulations.

## Supplementary information

Supplementary Information.

Supplementary Information.

Supplementary Information.
