# Supplementary material for: Nanoscale 3D quantitative imaging of 1.88 Ga Gunflint microfossils reveals novel insights into taphonomic and biogenic characters
Source: Sci Rep. 2020 May 18;10:8163. doi: 10.1038/s41598-020-65176-w (PMC7235231; doi:10.1038/s41598-020-65176-w)
Supplement: Supplementary file 3 — Supplementary Information. [file 41598_2020_65176_MOESM3_ESM.docx]

**Title:** Nanoscale 3D quantitative imaging of 1.88 Ga Gunflint microfossils reveals novel insights into taphonomic and biogenic characters

**﻿**

**Maldanis, L.**^[[1]](#footnote-2)^**^,^**^[[2]](#footnote-3)^ **^,*^; Hickman-Lewis, K.**^[[3]](#footnote-4)^**^,^**^[[4]](#footnote-5)^**; Verezhak, M.**^[[5]](#footnote-6)^**; Gueriau, P.**^[[6]](#footnote-7)^**^,^** **^﻿^**†**; Guizar-Sicairos, M.** ^5^**; Jaqueto, P.**^[[7]](#footnote-8)^**; Trindade, R.I.F.**^7^**; Rossi, A.L.**^[[8]](#footnote-9)^**; Berenguer, F.**^[[9]](#footnote-10)^**; Westall, F.**^3^**; Bertrand, L.**^6,9^**; Galante, D. 1**

**Supplementary Figures**


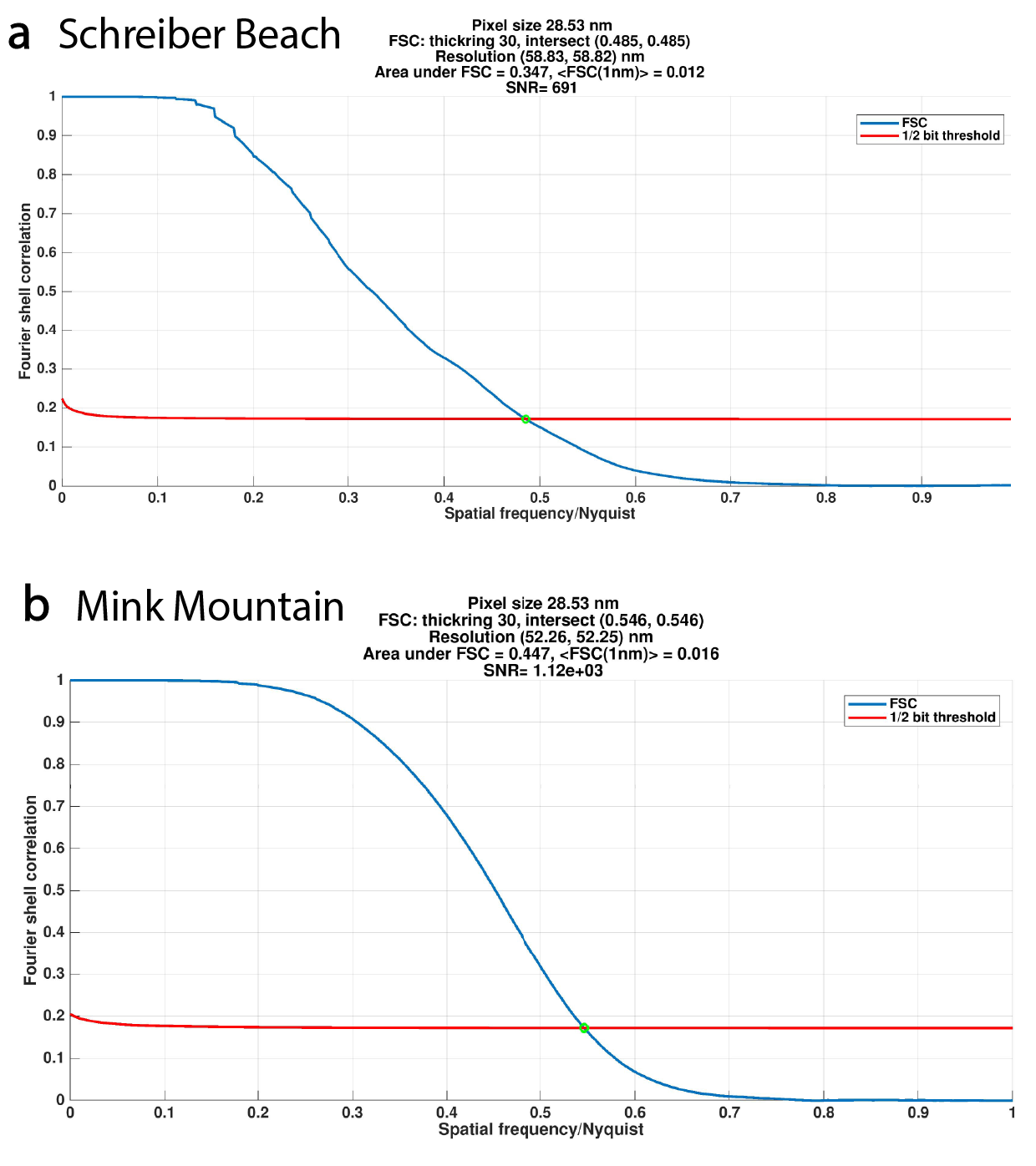


**Figure S1. Resolution estimation by Fourier shell correlation (FSC) yielding a three-dimensional resolution of 59 nm for Schreiber Beach and 52 nm for Mink Mountain.** The FSC curve is calculated between two independent 3D reconstructions, each from half of the data. The point in which the FSC curve intersects the half-bit threshold curve determines the effective half-pitch spatial resolution (21, 42, 48).


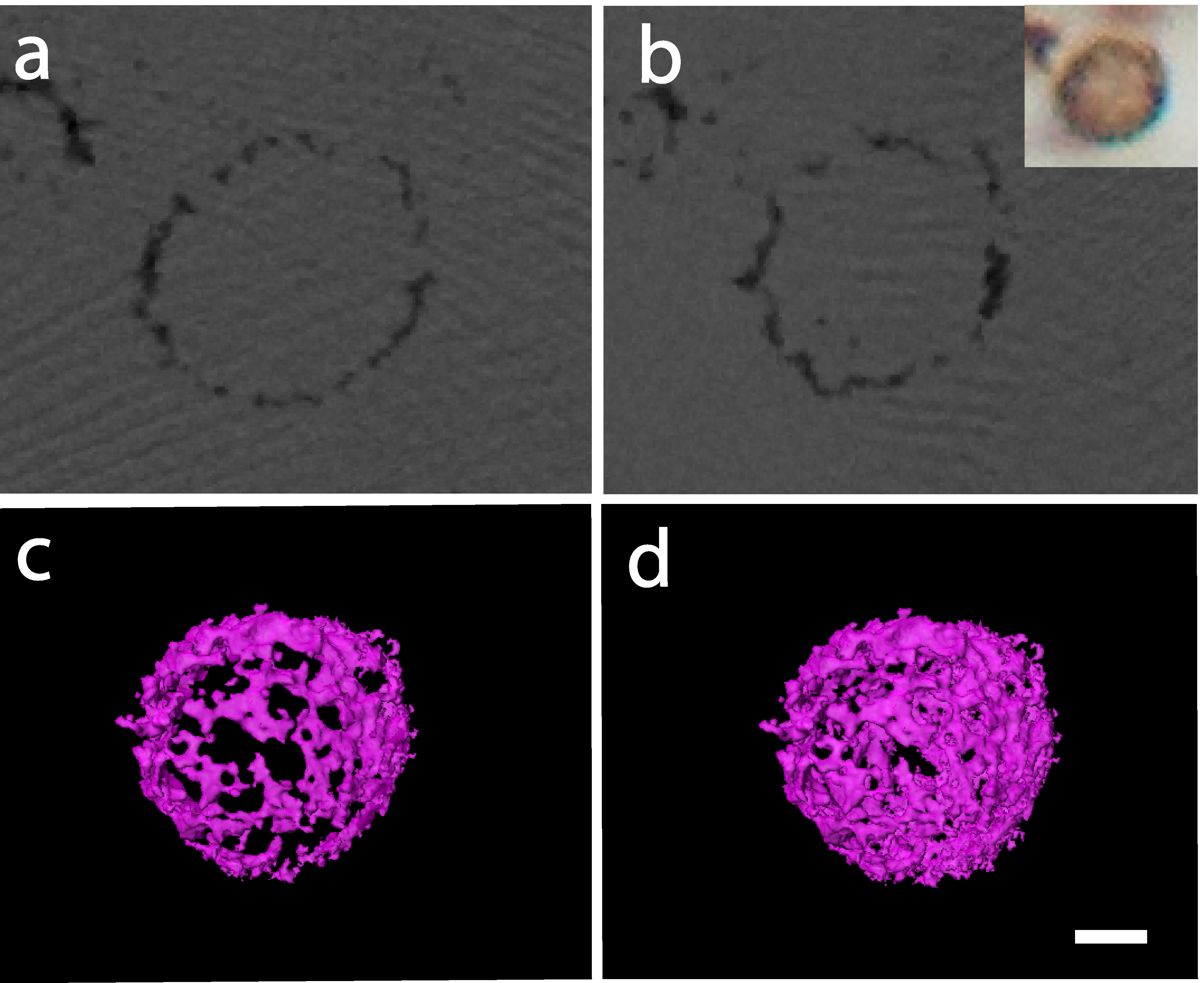


**Figure S2. Microfossil in Schreiber Beach sample identified as *Huroniospora****.* **a,b,** 2D virtual tomographic slices showing carbonaceous cell walls with a saw-tooth pattern resulting from taphonomic alteration*.* **c,** 3D rendering after removal of the structures in the back of the fossil to ease observation of cell wall structure. **d,** 3D view of the complete specimen. Scalebar 1 μm.


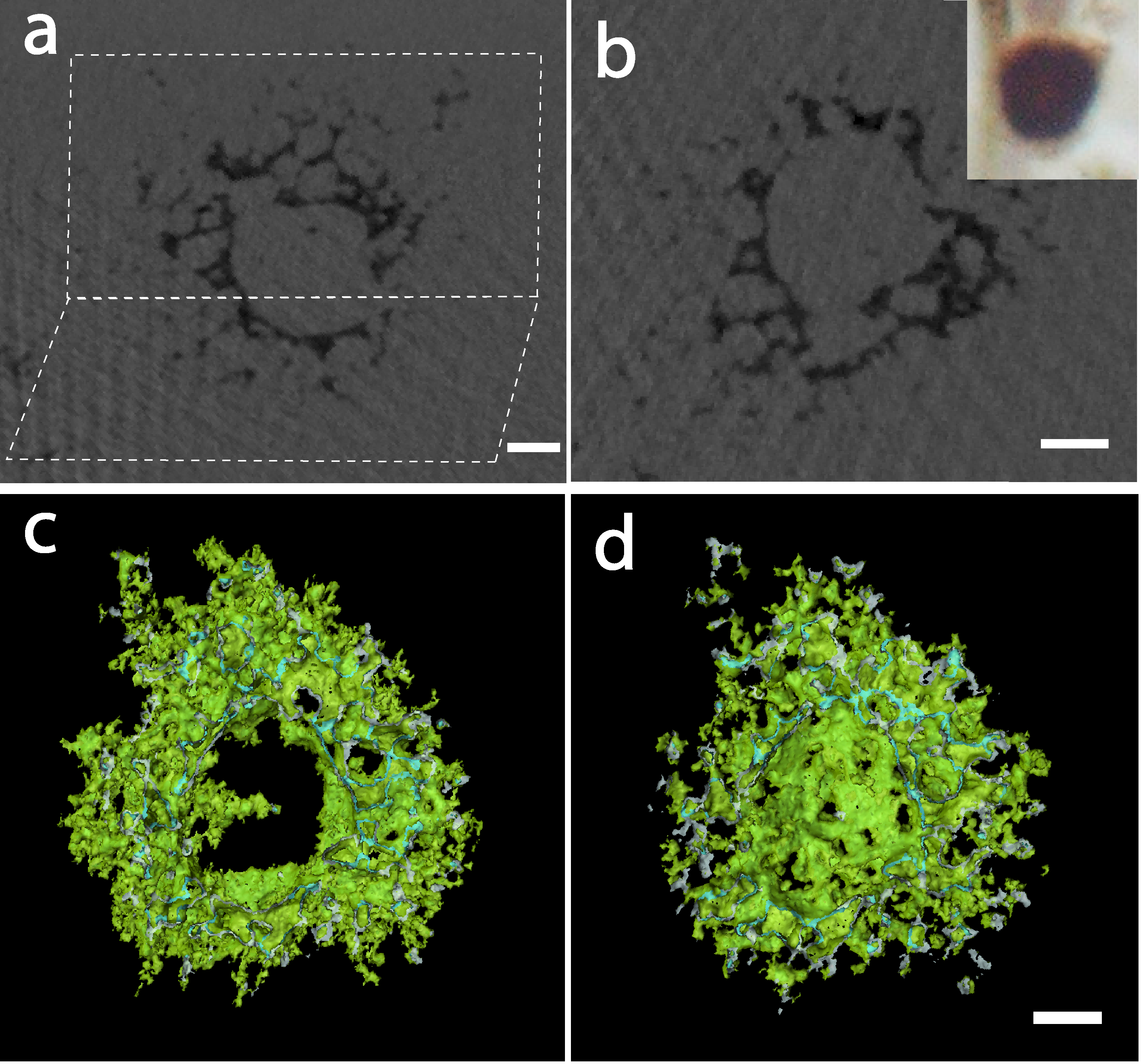


**Figure S3. Microfossil with a structural pattern similar to *Eosphaera.*** The outer margin of the cells is characterized by large, distinct spheroidal objects with diameters approximately 20-25% of that of the main body of the cell, morphologically most consistent with *Eosphaera*. **a,** intersection of 2 tomographic images in different planes (indicated by the dashed lines) showing the presence of tubercular small spheroids of different sizes in both planes surrounding a large central spheroid. **b**, detail of the distribution of tubercular spheroids surrounding a larger spheroid and optical micrography (upper left) showing the same pattern with visible light. **c**, 3D rendering of a section of the specimen showing its hollow aspect forming a large spheroid. **d**, 3D rendering of the complementary part presented in **c** showing the “bottom” of the specimen, i.e., the closing of the spheroid. Scalebar 1μm.


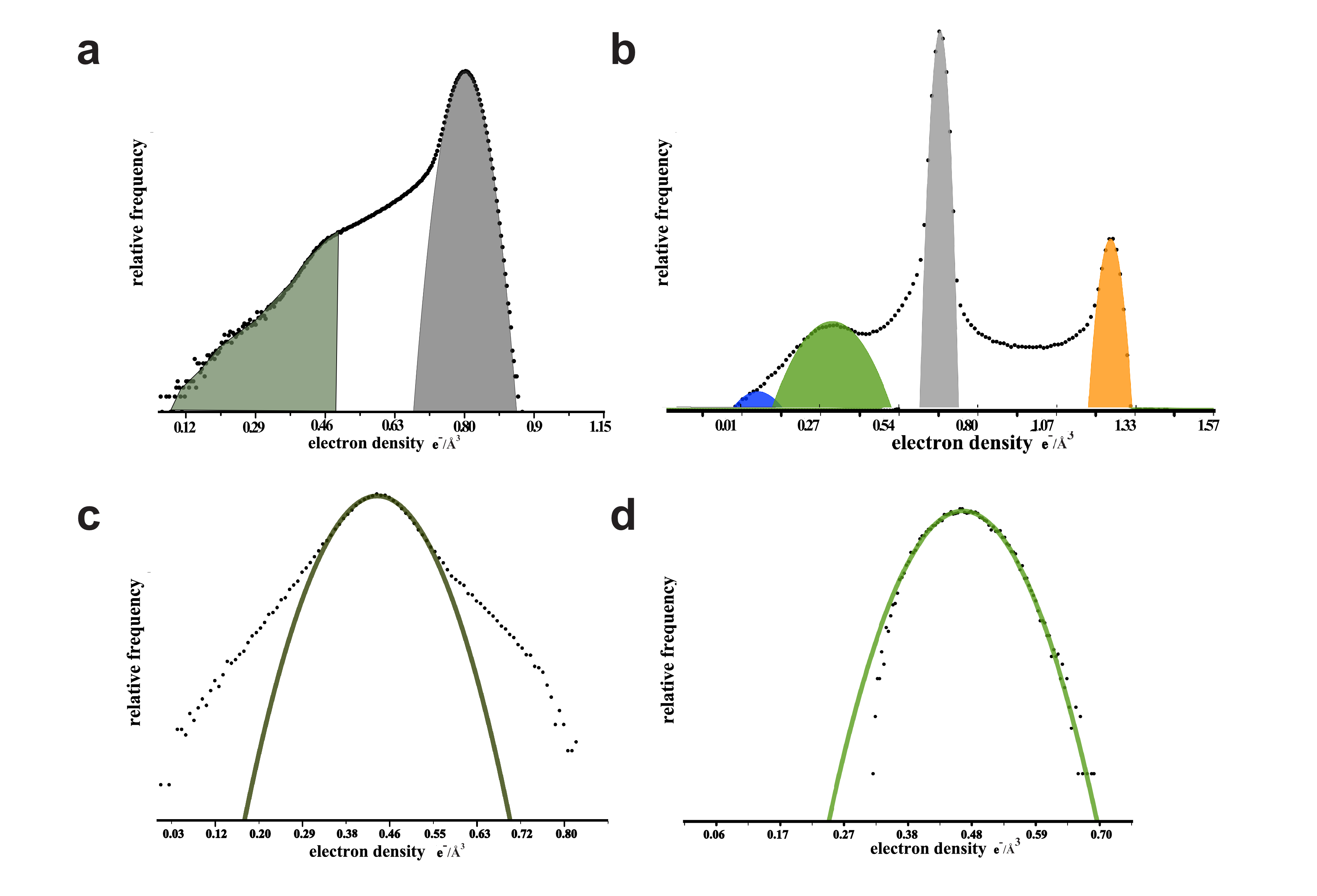


**Figure S4. Histogram of pixel intensity (electron density) of the sample from Schreiber beach (a,c) and Mink Mountain (b,d), in logarithmic scale. a, b,** Total histogram of the samples. In the Schreiber Beach sample (**a),** due to the small dimensions of the carbonaceous structures (green) a strong partial volume effect with the contribution of the surrounding silica matrix (grey) is observed, instead of a normal distribution. A similar effect can be seen in the Mink Mountain sample (b) between the kerogen (green) and the non-solid material phase within it (blue). **c**, Schreiber Beach and **d,** Mink mountain histograms of carbonaceous material after segmentation with their respective fitted gaussians (green).

**
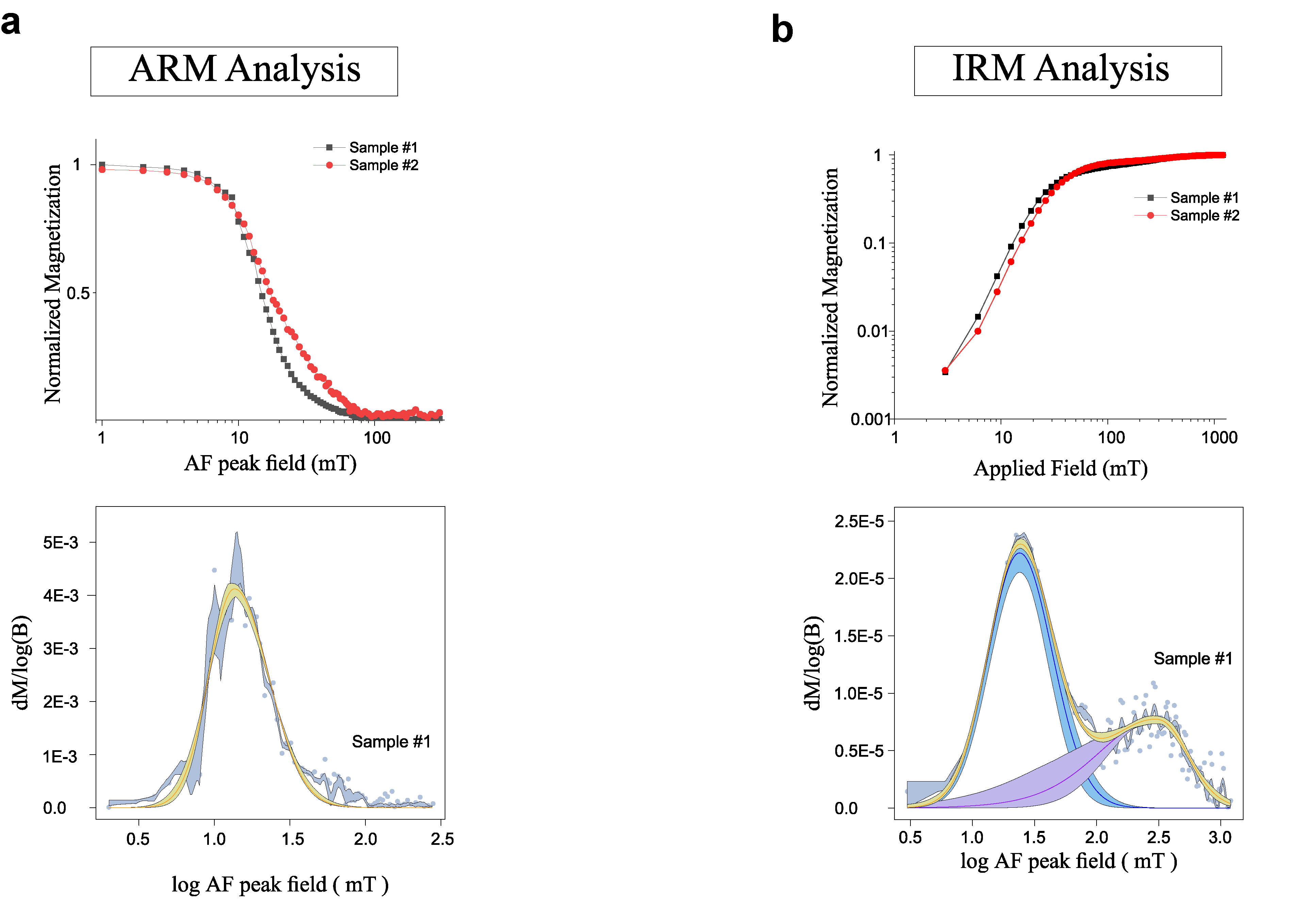
**

**Figure S5. Curves of remanence acquisition at room-temperature on two sections from the same sample of Mink Mountain analyzed by PXCT. a,** Anhysteretic remanent magnetization (ARM) demagnetization curves (upper) and the coercivity spectra with adjusted skewed generalized Gaussian function (51) (lower), indicating the presence of a low-coercivity phase (~20 mT, log_10_ value of 1.3, as seen in graph) interpreted as maghemite. **b,** Isothermal remanent magnetization (IRM) acquisition curves (upper), and their respective coercivity spectra (lower) indicating two magnetic components (~24 mT and ~343 mT, log_10_ values of 1.3 and 2.5), with the low-coercivity phase interpreted as maghemite and/or partially oxidized magnetite, the high-coercivity phase interpreted as hematite.

**
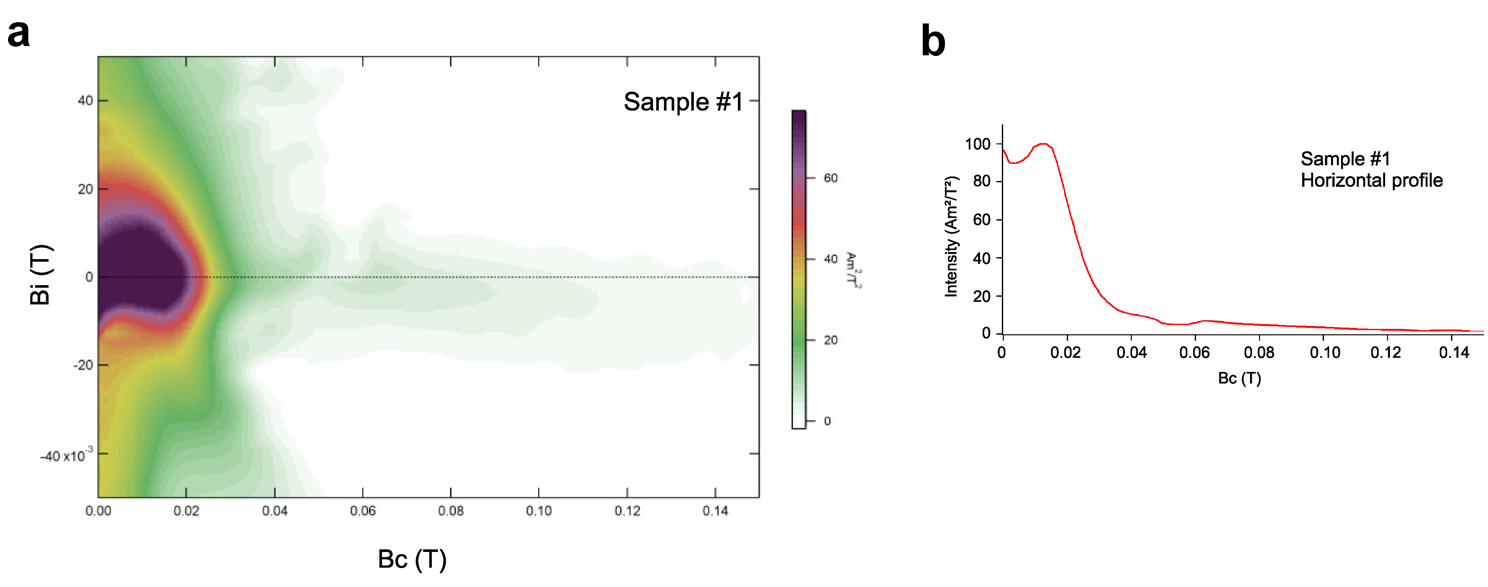
 Figure S6. First-order reversal curve (FORC) diagram of one section of the same sample of Mink Mountain analyzed by PXCT. a,** FORC diagrams contour map of the mixed partial second derivative from a class of partial magnetic hysteresis, with Bc (x-axis) representing the coercivity, and Bi (y-axis) representing the interaction. **b,** Profile along the Bi = 0 line. The FORC is typical of pseudo-single domain particles (52) with the coercivity between 10 mT and 20 mT, (lower than 40 mT, typical of single domain particles (53)) which for maghemite range it is between 1 and 20 μm (54-55).





**Figure S7. Raman point spectra of filament in the Mink Mountain locality sample adjacent to the filaments sampled for PXCT analysis.** The bands (227, 247, 294 412, 499, 612 and 1319 cm-^1^) are characteristic of hematite and quartz is identified by the band at 465 cm-^1^. The kerogen D band is not visible due to overlap with the hematite 2LO band at 1319 cm-^1^, but the G band can be seen at 1542 cm-^1^. Bands of maghemite and magnetite are not observed (35-37).


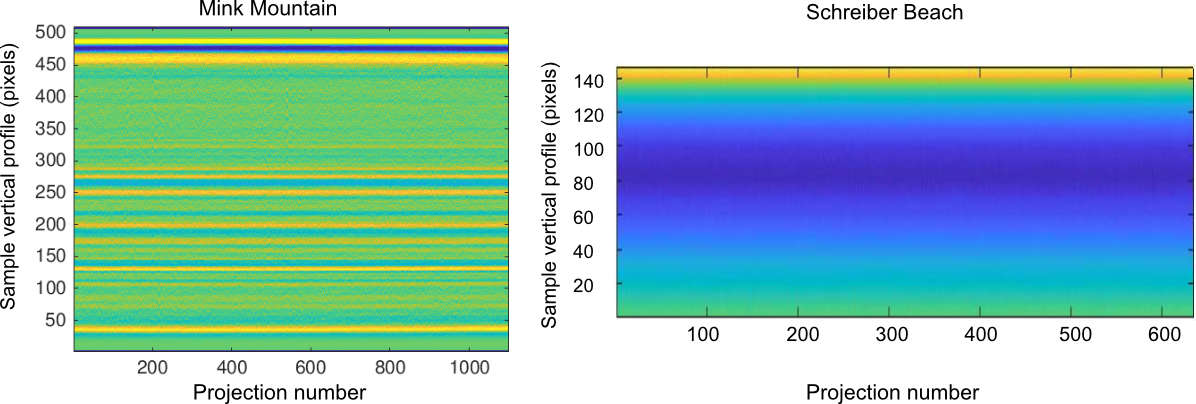


**Figure S8. Profile of the sample of Mink Mountain after the vertical mass fluctuations alignment** (2)**.** The vertical sample mass profile is constant along the 1100 projections. Radiation damage would result in a modification of this sample mass vertical profile, which is not observed here.


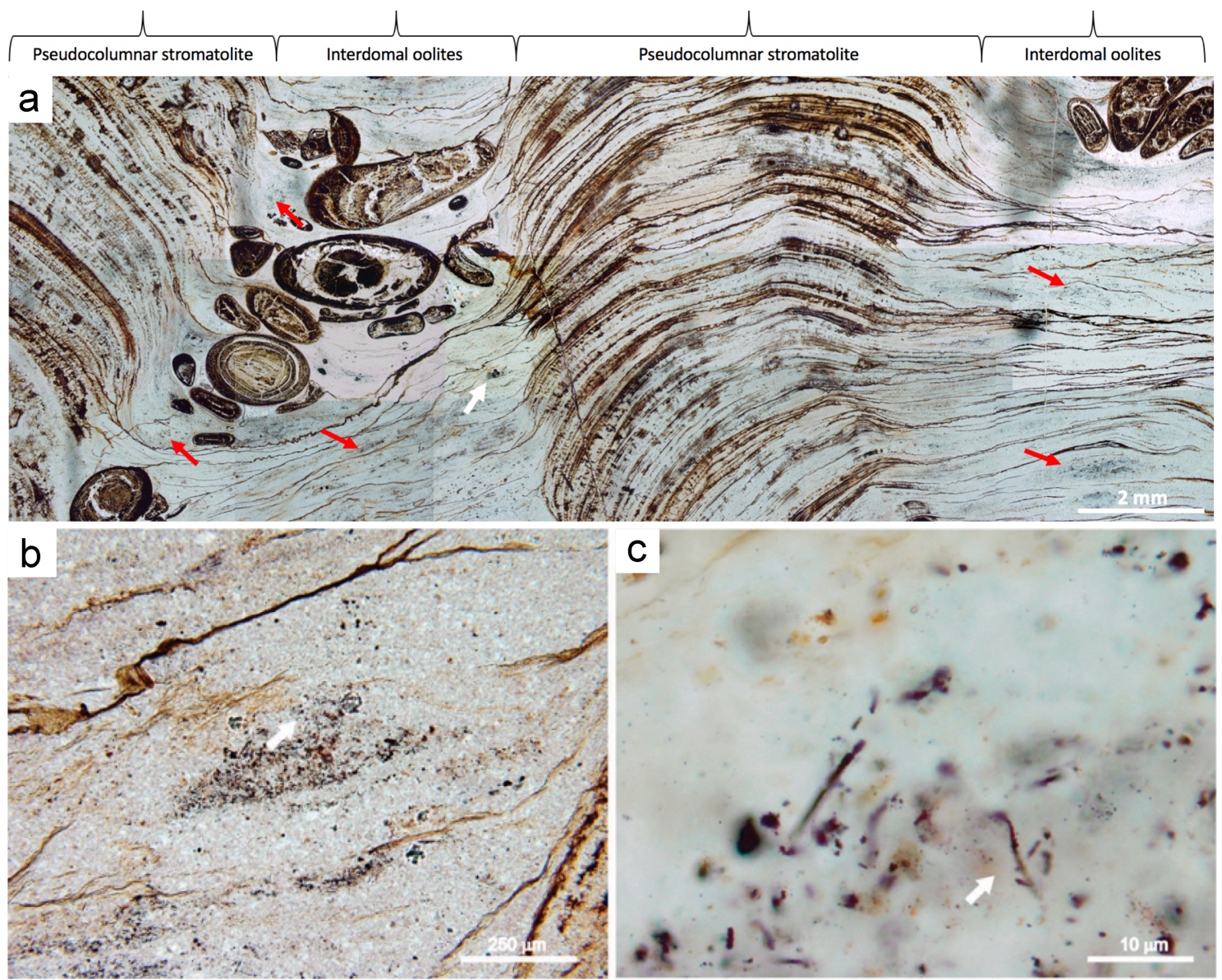


**Figure S9. Petrographic characterisation and sample selection (Mink Mountain) from within the limb of a pseudocolumnar stromatolite**. **a,** Macro-scale structure in thin section, comprising irregularly spaced stromatolitic columnar structures separated by troughs in which spherical oolites accumulate. Red arrows indicate microfossil-rich zones. The area marked with a white arrow is magnified in **b,c** and indicates the location from where the studied sample was selected. **b** Microfossil-rich region from which a pillar was extracted for analysis by PXCT. **c,** Photomicrographs showing the filamentous *Gunflintia sp.* Barghoorn microfossils (arrowed) studied herein by PXCT (see Fig. 1).

**Supplementary Videos**

**Video S1. Detailed view of Mink Mountain specimens.** Overview of sample volume, tomographic slices and 3D rendering of the microfossils immersed in the rock. The kerogenous material (rendered in green) has an irregular aspect, with inner fractures (rendered in blue) from its geochemical maturation. The maghemite crystals (rendered in orange) have anhedral and euhedral morphologies, some displaying cubic and octahedral morphologies.

**Video S2. Detailed view of Schreiber beach specimens**. Overview of the tomographic slices showing different morphologies within the sample volume and 3D rendering of the microfossils immersed in the rock. All the microfossils are composed of only kerogenous material outlining thin and discontinuous cell walls.

**Supplementary References**

51. Maxbauer, D. P., Feinberg, J. M. & Fox, D. L. MAX UnMix: A web application for unmixing magnetic coercivity distributions. *Computers and Geosciences* **95**, 140–145 (2016).

52. Roberts, A. P., Pike, C. R. & Verosub, K. L. First-order reversal curve diagrams: A new tool for characterizing the magnetic properties of natural samples. *Journal of Geophysical Research: Solid Earth* **105**, 28461–28475 (2000).

53. Roberts, A. P., Heslop, D., Zhao, X. & Pike, C. R. Understanding fine magnetic particle systems through use of first-order reversal curve diagrams. *Reviews of Geophysics* **52**, 557–602 (2014).

54. Roberts, A. P. *et al.* Resolving the Origin of Pseudo-Single Domain Magnetic Behavior. *Journal of Geophysical Research: Solid Earth* **122**, 9534–9558 (2017).

55. Dunlop, D. J. Theory and application of the Day plot ( M rs / M s versus H cr / H c ) 2. Application to data for rocks, sediments, and soils . *Journal of Geophysical Research* **107**, 1–15 (2002).

1. A**uthor Affiliation:**

   Brazilian Synchrotron Light Laboratory (LNLS), ﻿Brazilian Center for Research in Energy and Materials (CNPEM), ﻿Av. Giuseppe Maximo Scolfaro, 10000, 13083-100, Campinas, Brazil [↑](#footnote-ref-2)
2. Institute of Physics of São Carlos, University of São Paulo, ﻿Av. Trabalhador são-carlense, 400, 13566-590, São Carlos, Brazil [↑](#footnote-ref-3)
3. Centre de Biophysique Moléculaire, CNRS, Rue Charles Sadron, 45071, Orléans, France [↑](#footnote-ref-4)
4. Dipartimento di Scienze Biologiche, Geologiche e Ambientali (BiGeA), Università di Bologna, Piazza di Porta S. Donato, 1, 40126 Bologna, Italy [↑](#footnote-ref-5)
5. ﻿Paul Scherrer Institut, Forschungsstrasse 111, 5232 Villigen, Switzerland [↑](#footnote-ref-6)
6. IPANEMA, CNRS, Ministère de la Culture, UVSQ, Université Paris-Saclay, site du synchrotron SOLEIL, L’Orme des Merisiers, BP 48 Saint-Aubin, 91192, Gif-sur-Yvette, France [↑](#footnote-ref-7)
7. Department of Geophysics, Institute of Astronomy, Geophysics and Atmospheric Sciences, University of São Paulo, Rua do Matão, 1226, 05508-090, São Paulo, Brazil [↑](#footnote-ref-8)
8. Brazilian Center for Research in Physics (CBPF), R. Dr. Xavier Sigaud, 150, 22290-180, Rio de Janeiro, Brazil [↑](#footnote-ref-9)
9. Synchrotron Soleil, Saint-Aubin, L’Orme des Merisiers, BP 48 Saint-Aubin, 91192, Gif-sur-Yvette, France

   **^*^** Current address: ISterre, UGA, CNRS, Observatoire des Sciences de l’Univers, CS 40700, 38058, Grenoble, France.

   †Current address: Institute of Earth Sciences (ISTE), University of Lausanne, Lausanne, Switzerland

   **Corresponding Author:**

   Lara Maldanis

   [lara.maldanis@univ-grenoble-alpes.fr](mailto:laramcp@hotmail.com) [↑](#footnote-ref-10)
